# Leprosy Drug Resistance Surveillance in Colombia: The Experience of a Sentinel Country

**DOI:** 10.1371/journal.pntd.0005041

**Published:** 2016-10-05

**Authors:** Camilo Beltrán-Alzate, Fernando López Díaz, Marcela Romero-Montoya, Rama Sakamuri, Wei Li, Miyako Kimura, Patrick Brennan, Nora Cardona-Castro

**Affiliations:** 1 Instituto Colombiano de Medicina Tropical–Universidad CES Sabaneta, Antioquia, Colombia; 2 Sanatorio de Agua de Dios, Agua de Dios Cundinamarca, Colombia; 3 Department of Microbiology, Immunology and Pathology, Colorado State University, Fort Collins, Colorado, United States of America; 4 Facultad de Medicina. Universidad CES, Medellín, Colombia; Fondation Raoul Follereau, FRANCE

## Abstract

An active search for *Mycobacterium leprae* drug resistance was carried out, 243 multibacillary patients from endemic regions of Colombia were included from 2004 to 2013 in a surveillance program. This program was a World Health Organization initiative for drug resistance surveillance in leprosy, where Colombia is a sentinel country. *M. leprae* DNA from slit skin smear and/or skin biopsy samples was amplified and sequenced to identify mutations in the drug resistance determining region (DRDR) in *rpoB*, *folP1*, *gyrA*, and *gyrB*, the genes responsible for rifampicin, dapsone and ofloxacin drug-resistance, respectively. Three isolates exhibited mutations in the DRDR *rpoB* gene (Asp441Tyr, Ser456Leu, Ser458Met), two in the DRDR *folP1* gene (Thr53Ala, Pro55Leu), and one isolate exhibited mutations in both DRDR *rpoB* (Ser456Met) and DRDR *folP1* (Pro55Leu), suggesting multidrug resistance. One isolate had a double mutation in *folP1* (Thr53Ala and Thr88Pro). Also, we detected mutations outside of DRDR that required in vivo evaluation of their association or not with drug resistance: *rpoB* Arg505Trp, *folP1* Asp91His, Arg94Trp, and Thr88Pro, and *gyrA* Ala107Leu. Seventy percent of *M*. *leprae* mutations were related to drug resistance and were isolated from relapsed patients; the likelihood of relapse was significantly associated with the presence of confirmed resistance mutations (OR range 20.1–88.7, p < 0.05). Five of these relapsed patients received dapsone monotherapy as a primary treatment. In summary, the current study calls attention to *M*. *leprae* resistance in Colombia, especially the significant association between confirmed resistance mutations and relapse in leprosy patients. A high frequency of DRDR mutations for rifampicin was seen in a region where dapsone monotherapy was used extensively.

## Author Summary

*Mycobacterium leprae* drug resistance is cause of surveillance due to the increase of leprosy relapsed cases. World Health Organization initiative for drug resistance surveillance in leprosy included Colombia, a country considered in post-elimination stage, as a sentinel country. During 10 years (2004–20013) an active search for *M*. *leprae* drug resistance was carried out in volunteer patients (each of whom signed a consent form) recruited from a convenience sample of patients diagnosed with leprosy in fourteen departments of Colombia: Amazonas, Antioquia, Atlántico, Bolívar, Caquetá, Cesar, Cundinamarca, Chocó, Huila, Magdalena, Norte de Santander, Santander, Tolima, and Valle. 243 multibacillary patients in various stages of multi drug therapy (MDT) were enrolled with the aim to search for primary and secondary *M*. *leprae* drug resistance: 33 new patients before treatment, 136 currently undergoing MDT with not clinical improvement of lesions after three months or more of MDT, 36 post-MDT with positive bacillary index (BI) persistence, 4 non-adherent to MDT, and 34 relapsed cases. *M. leprae* DNA from patient´s samples was tested to identify mutations in the drug resistance determining region (DRDR) in *rpoB*, *folP1*, *gyr*A, and *gyrB*, the genes responsible for rifampicin, dapsone and ofloxacin drug-resistance, respectively. We obtained isolates that exhibited mutations in the DRDR, three in *rpoB* gene, two in *folP1* gene, and one isolate exhibited mutations in both *rpoB* and *folP1*, suggesting multidrug resistance. One isolate had a double mutation in *folP1*. Also, we detected mutations outside of DRDR that required in vivo test validation. 70% of *M*. *leprae* confirmed resistance mutations were isolated from relapsed patients (OR range 20.1–88.7, p < 0.05). Five of these relapsed patients received dapsone monotherapy as a primary treatment. The current study calls attention to *M*. *leprae* resistance in Colombia, especially the significant association between confirmed resistance mutations and relapsed in leprosy patients.

## Introduction

Leprosy is a chronic infectious disease caused by *M. leprae* that results in disfigurement, exclusion from society and, often, in or from poverty [1]. Prior to the 1940s, leprosy was considered an untreatable disease. However, in the early 1940s, diamino-diphenyl sulfone (DDS; also known as dapsone), was shown to successfully treat leprosy. At that time, dapsone was used for treatment of leprosy until lesions were cured or until the bacillary index (BI) became negative, a process that took decades [2, 3] and one that often resulted in patients relapsing after the termination of treatment [3]. In the 1980’s, the World Health Organization (WHO) recommended dapsone, rifampicin and clofazimine as the primary agents for multidrug therapy [4]. The frequency of clinical relapse due to dapsone resistance after monotherapy, is higher than the results of dapsone resistance tests in mouse foot pad since few strains exhibited full resistance [5]. Nevertheless, resistance to low levels of dapsone is detected in mouse footpads, indicative of inadequate treatment. Dapsone is a bacteriostatic antibiotic and the persistence of bacilli may be one cause of relapse in dapsone monotherapy [6].

WHO, concerned with clinical relapses, emergence, and transmission of organisms with low levels of resistance to dapsone, recommended the addition of rifampicin, a bactericidal drug taken from the TB treatment stock [4]. Thus, multidrug therapy (MDT) for leprosy was initiated in the early 1980s [3,4]. To treat paucibacillary leprosy (PB), the MDT combination is dapsone and rifampicin. To treat multibacillary (MB) leprosy, clofazimine was included as a third drug [4,6]. Currently, WHO recommends an MDT duration of 1 year for MB leprosy and 6 months for PB leprosy. Globally, these guidelines apply to more than a million patients, having been adapted to a variety of resource-constrained settings over the past decades [3].

In Colombia, MDT was introduced in 1985 when the prevalence of leprosy was 5.5 cases per 10,000 people. In 1997, Colombia achieved the goal of elimination proposed by WHO: a prevalence of less than 1/10,000. Although leprosy treatment in Colombia is based on WHO guidelines, some modifications are made according to the criteria of clinicians [7,8]. For example, the duration of treatment for PB patients may be extended for one year, and in the case of MB the treatment, up to 2 years, if lesions persist and the Bacillary index (BI) is positive [7,9]. Leprosy resistance in Colombia has been associated with relapses, especially in patients undergoing dapsone monotherapy [10, 11].

When the molecular targets for DDS and rifampicin were identified, drug resistant bacterial strains with high resistance in mouse footpad systems were found to have mutations in genes encoding dihydropteroate synthase (*dhps* or *folP1*) and RNA polymerase B (*rpoB*) [12–15]. However, drug resistant strains exist that do not have mutations in known genetic targets [3, 4]. While mutations that confer low levels of resistance have not been identified, such low-level resistance is seen in wild-type strains [4, 13]. The mechanism of clofazimine resistance has been under investigation for many years. Recently, it was shown that clofazimine is a substrate for bacterial type-2 NADH reductase [16]. However, mutations for genes involved in this redox process have not been ascribed to clinical clofazimine resistance [16]. MDT treatment of new leprosy cases is not routinely accompanied by monitoring for primary or secondary drug resistance [3, 6]; surveillance and retreatment of dapsone monotherapy in patients undergoing MDT is practiced in only in a few settings [15,17]. Treatment of returning patients with additional rounds of MDT, without knowledge of eventual underlying drug resistance, is the default course of action in leprosy control programs [7,18,19]. Unfortunately, dwindling resources for leprosy does not allow for individualized treatment regimens and long-term follow-ups [7]. Research has shown that patients with a high pre-MDT bacteriological index (>4) are at risk of relapsing after MDT, with relapses occurring 6–16 years after the completion of treatment [12]. An earlier study showed that a high pre-MDT bacterial index is also a risk factor for relapse. Relapses were not due to drug resistance, as partial and full dapsone resistance was detected in only 3 of 18 relapses [20].

Thus, distinguishing relapses due to incomplete treatment, persistent bacilli, drug resistance and re-infection are challenges for clinicians and health care workers. The definition of relapse differs for those undergoing dapsone monotherapy and those receiving fixed, 2 year MDT (and the now shortened 1 year MDT) [19]. Reduction of BI is a slow process, dropping only one unit each year with MDT. Therefore, no proper end point for the completion of treatment exists and BI determination is not a universally practiced standard of care [19].

For better understanding of the extent of primary and secondary drug resistance, researchers worldwide have independently used molecular epidemiological approaches. In 2006, WHO called for leprosy drug resistance surveillance (DRS) in several sentinel countries [18, 19]. This goal of this study was to report the DRDR mutations in leprosy patients from Colombia from a 10 year WHO recommended surveillance program.

## Methods

### Patients

This molecular epidemiology survey was performed using 243 individual *M*. *leprae* DNA samples from Colombian leprosy patients from 2004 to 2013. Volunteer patients (each of whom signed a consent form) were recruited from a convenience sample of patients diagnosed with leprosy in fourteen departments of Colombia: Amazonas, Antioquia, Atlántico, Bolívar, Caquetá, Cesar, Chocó, Cundinamarca, Huila, Magdalena, Norte de Santander, Santander, Tolima and Valle. Patients, in various stages of treatment were enrolled with the aim to search for primary and secondary *M*. *leprae* drug resistance and included 33 new patients before treatment,136 currently undergoing treatment with not clinical improvement of lesions after three months of treatment, 36 post-treatment with positive bacillary index (BI) persistence, 4 non-adherent to treatment, and 34 relapse cases.

Bacterial DNA was extracted from skin biopsies using the Dneasy Blood & Tissue Qiagen kit (Invitrogen) according to the manufacturer’s protocol [21,22]. Slit skin samples were collected from four different sites, with an emphasis on active lesions. Extracted DNA was stored at -20°C until processing.

### Ethical considerations

The ethical committee of the Instituto Colombiano de Medicina Tropical–Universidad CES approved this project as the minimal risk, all the data analyzed were anonymized.

### Amplification and sequencing analysis

Multiplex PCR was performed using primers suggested by WHO to amplify the DRDR regions of the *rpoB*, *folP1*, *gyrA* and *gyrB* genes [19]. PCR reactions were performed in 25 μl reaction volumes: 2.50 μl of 10X Amplitaq Gold PCR Mastermix (Applied Biosystems, USA), 2.50 μl of MgCl2 (25 mM), 1.25 μl of each primer (0.3 mM), 8μl of sterile, deionized water and 2.0 μl extracted DNA. A PIKO thermocycler (Finnzymes Instruments) was employed with the following amplification conditions: activation of AmpliTaq Gold at 95°C for 5 min, 40 cycles at 95°C for 15 sec, 60°C for 15 sec, 72°C for 60 sec, and a final elongation at 72°C for 7 min.

For each amplification, 2 μl of purified *M*. *leprae* DNA (reference strain 4264) was used as a positive control. 2 μl of RNAse-free ultrapure water and 2 μl of DNA of *Salmonella enteritidis* (ICMT-99 strain) were each used as negative controls. DNA products were electrophoresed on agarose gel in 2.5% agarose gel inTE buffer and stained with ethidium bromide. A 20 pb molecular weight marker was used to determine amplicons size (Biorad, Cat No. 1708351).

PCR products were sequenced using on an ABI PRISM 3130xl genetic analyzer (Applied Bio systems) at the Colorado State University core sequencing facility. Chromatograms were analyzed with Chromas lite software [23] and compared with *M*. *leprae* reference sequences of antibiotic sensitive strains (in the leproma database http://genolist.pasteur.fr/Leproma/) by BLAST, as suggested by WHO [24].

### Statistical analysis

The odds ratio and 95% CI of the relationship between likelihood of relapse and the presence of confirmed resistance mutations was calculated and significance assessed by Chi square test. P < 0.05 was considered significant.

## Results

**Fig 1:**
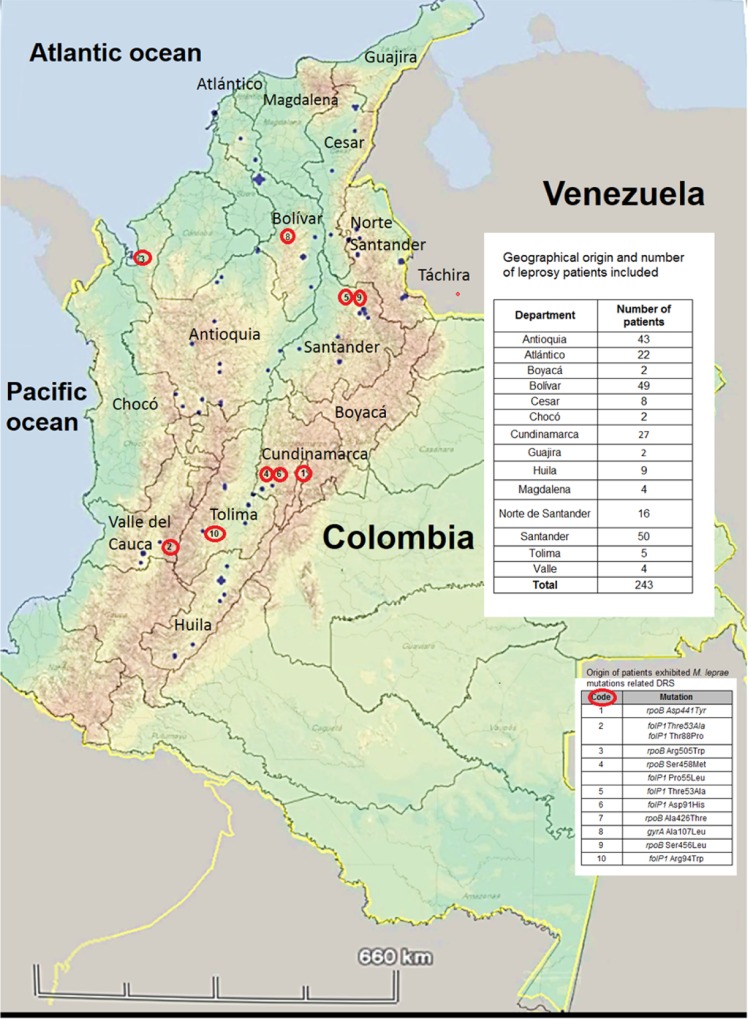
Geographical origin of leprosy patients that exhibits *M*. *leprae* drug resistance related mutations.

The geographical distribution of patients included in this study and the origin of *M*. *leprae* DRDR mutations are shown in Fig 1. The Andean region had 50% of *M*. *leprae* DRDR mutations. Of note, within this Andean region are the departments of Cundinamarca and Santander where two existing Colombian leprosaria, Agua de Dios and Contratación, are located.

The distribution of samples according to patient origin, gender and age is summarized in Table 1. Overall, the male to female ratio was 2.45:1; 72.2% of patients were male. The mean age was 52 years with a range of 9 to 91 years.

**Table 1:** Demographic, therapeutic, and clinical characteristics of patients involved in the survey.

Department/ Incidence 2013●	N =	Males	Females	Treatment				Leprosy reaction	Relapse	
				Pre Tto*	Under Tto**	Post Tto***	Non Adh #			
									After DDS†	After MDT‡
Antioquia/0.19	43	35	8	15/43	16/43	9/43	0	15/43	3/43	0
Atlántico/0.92	22	19	3	1/22	21/22	0	0	3/22	1/22	1/22
Boyacá/0.39	2	1	1	0	2/2	0	0	0	0	0
Bolívar/2.98	49	35	14	6/49	29/49	11	1/49	0	2/49	3/49
Cesar/1.99	8	6	2	0	4/8	1/8	3/8	0	1/8	0
Chocó/0.20	2	1	1	2/2	0	0	0	0	0	2/2
Cundinamarca/0.3	27	19	8	5/27	10/27	4/27	9/27	1/27	9/27	4/27
Guajira/0.0	2	0	2	0	2/2	0	0	0	0	0
Huila/2.48	9	7	2	5/9		3/9	1/9	0	0	0
Magdalena/2.87	4	4	0	1/4	2/4	0	¼	0	0	0
Norte de Santander/3.90	16	13	3	1/16	3/16	10/16	2/16	1/16	0	0
Santander/2.74	50	38	12	3/50	39/50	5/50	3/50	7/50	1/50	2/50
Tolima/0.85	5	4	1	0	4/5	1/5	0	3/5	0	1/5
Valle/0.79	4	2	2	0	4/4	0	0	0	0	1
**Total**	243	184	59	45/243	131/243	44/243	20/243	30/243	17/243	13/243

* Before leprosy treatment.

** Under treatment.

*** After treatment has ended.

# Non-adherence to treatment.

† Dapsone monotherapy.

‡ Multi drug therapy.

● Cases/100.000 reported in 2013.

Positive PCR and successful sequencing were not obtained in all cases and could be due to DNA as a consequence of treatment. Positive PCR and successful DNA amplification were exhibited for *rpoB* (n = 134/243), *folP1* (n = 215/243), *gyrA* (n = 200/243) and *gyrB* (n = 184/243).

Sequences showed that three isolates exhibited mutations in DRDR *rpoB* gene (Asp441Tyr, Ser456Leu, Ser458Met), and two in DRDR *folP1* gene (Thr53Ala, Pro55Leu). One isolate exhibited mutations in both DRDR *rpoB* (Ser456Met) and DRDR *folP1* (Pro55Leu). This result in this isolate suggested multidrug resistance (Table 2).

**Table 2:** Clinical characteristics of patients exhibited *M*. *leprae* DRDR mutations.

Date of report/ Code*	Age	Sex	Lesions	Number of lesions	Bacillary Index	Mutations	Date Past Diagnosis/ Therapy	Actual Diagnosis/ Therapy
2006/ Code 1	66	M	Lepromas in arms and legs	15	3+	*rpoB* Asp441Tyr+	1960-LL/MNT**	Relapse LL/MDT
2008/ Code 2	50	M	Nodules, skin infiltrates in face, thorax, abdomen, legs	20	1+	*folP1* Thre53Ala+ *folP1* Thr88Pro‡	1976-LL/MNT	Relapse LL/MDT
2010/ Code 3	66	M	Type II reaction, anesthesia, paresthesia, hypo and hyper pigmented plaques in legs and arms, thorax, back, leonine fascies, madarosis, nodules, infiltrates	>25	2+	*rpoB* Arg505Trp+	1990-LL/MDT***	Relapse LL/MDT
2006/ Code 4	58	M	Nodules in arms, legs, loss of sensitivity	7	1+	*rpoB* Ser458Me+t *folP1* Pro55Leu+	1972-LL/MNT 1983-LL/MDT 2003-Leprosy Reaction 2006-LL/MDT	Relapse LL/MDT
2008/ Code 5	42	M	Hypo and hyper-pigmented macula, skin infiltrates in arms, thorax, abdomen, legs since two years ago.	12	3+	*folP1* Thre53Ala+	Under treatment	LL/MDT
2006/ Code 6	52	M	Nasal septum affected, claw hands, ulnar nerve thick, skin patches, infiltration in arms, legs, since six months	10	2+	*folP1* Asp91His‡	1967-LL/MNT 1988-LL/MDT	Relapse LL/MDT
2011/ Code 7	81	M	Lesions, macules in superior limbs, back, thorax, generalized xerosis, bone reabsortion in right foot, madarosis, tibial ulcers	11	2+	*rpoB* Ala426Thre+	1990-LL/MDT	Relapse LL/MDT
2008/ Code 8	66	M	Macular lesions in ear lobes	2	1+	*gyrA* Ala107Leu‡	Under treatment	BL/MDT
2011/ Code 9	84	M	Macules, plaques	>5	5+	*rpoB* Ser456Leu+	1946-LL/MNT2011-LL/MDT	Relapse or Reinfection not confirmed/ MDT
2011/ Code 10	56	M	Leprosy reaction	>5	2+	*folP1* Arg94Trp‡	2010-LL/ MDT	Leprosy reaction/MDT

* Code of patients exhibiting *M*.*leprae* drug-resistance mutations is located in Fig 1.

** MNT monotherapy DDS.

*** Multidrug therapy.

+ DRDR: mutation inside Drug Resistance Determining Region.

‡ Mutation outside DRDR needs confirmation by foot pad mice test.

One isolate had a double mutation in *folP1*: Thr53Ala and Thr88Pro (outside of DRDR). Six mutations outside of DRDR that required an in vivo test for validation were found: *rpoB* Ala426Thre and Arg505Trp, *folP1* Asp91His, Arg94Trp, and Thr88Pro, and *gyrA* Ala107Leu (Table 2).

It is remarkable that all mutations found in DRDR for *rpoB* and *folP1* were detected in seven relapsed patients and in one patient currently undergoing treatment (Table 2).

Mutations found in *rpoB* (Ala426Thre, Asp441Tyr, Ser456Leu, Ser458Met, and Arg505Trp) were within, or close to, the DRDR found between nucleotides 432 to 458 [25–29]. The *folP1* mutations (Thr53Ala and Pro55Leu) were located in the DRDR [28, 30].

Further, we found isolates from three relapsed patients that exhibited mutations that will require further validation by mouse footpad method, in specialized laboratories—two isolates in *rpoB* and one isolate in *folP1*. The two mutations in *rpoB* (Ala426Thr and Arg505Trp) were close to the rifampicin resistance determining region located between nucleotides 432 and 458 [25–29]. The *folP1* mutation (Asp91His) was located outside the dapsone resistance determining region, at positions 53 and 55 [28, 30].

Regarding *gyrA*, no isolate displayed a mutation in the region determining fluoroquinolone resistance (nucleotides 89–91) [28–31]. However, one patient under treatment exhibited a mutation within *gyrA* (Ala107Leu) that also will require further validation by mouse footpad method, in specialized laboratories, for ofloxacin resistance.

In terms of frequency, five of the 134 cases with a positive amplification and DNA sequence (3.7%) had mutations in *rpoB*, five of 215 (2.32%) in *folP1*, and one of the 200 (0.5%) in *gyrA*. None contained a mutation in *gyrB*.

Four of 34 (11.8%) relapsed patients exhibited isolates with mutations in the DRDR *rpoB* and/or *folP1*. In contrast, one of 209 (0.47%) non-relapse patients contained isolates with mutations in DRDR *folP1*mutations.

Overall, the likelihood of relapse was significantly associated with the presence of confirmed resistance mutations: resistance to dapsone (OR 20.1 [95% CI 2.0–99.6], p = 0.01), resistance to rifampicin (OR 39.7 [95% CI 2.1–56.9], p = 0.01), and resistance to either dapsone or rifampicin (OR 88.7 [95% CI 10.5–747.3], p < 0.01).

## Discussion

In this report, drug resistant *M*. *leprae* strains from 243 patients at various leprosy clinical and treatment stages were determined. The study utilized a patient group assembled over a ten-year period. While the threshold for alarm over drug resistance to MDT, locally and globally, has not been well defined, we demonstrate that single and multi-drug resistant strains are detectable in Colombia, albeit at low frequencies [10, 11].

Of notable concern is the detection of a *folP1* mutation previously related with drug resistance in one patient under MDT that had not previously undergone DDS treatment, and in two others completing one and two treatments of MDT. Similarly, three patients had *rpoB* drug resistance mutations—one exposed to rifampicin during the first cycle of MDT and two treated with more than one cycle of MDT. The finding that one patient had dapsone and rifampicin resistance, combined with a previous report concerning two cases of rifampicin resistance [10], suggests that the rifampicin resistance is not trivial in Colombia, a country considered in the post-elimination stage of leprosy (prevalence <1/10,000). Despite the known presence of dapsone resistance mutations, relapsed patients are commonly retreated with an MDT regimen that contains dapsone.

We observed novel mutations in relapsed patients (Table 2) including *folP1* Thr88Pro, *rpoB* Ala426Thre, Arg505Trp, and *gyrA* Ala107Leu. Clinical evidence of relapse and mutations DRDR genes, can help the clinicians to choose alternative treatments to the patient, such as a combination of rifampicin, ofloxacin and minocycline [32]. However, identifying drug resistance in leprosy patients implies clinical, logistical and financial challenges. Further, the change of treatment is not a common clinical practice since drug resistance tests are not available in the routine activities of the leprosy control programs in Colombia, much less in the majority of endemic regions [7]. Also, alternative treatments are not freely available in the leprosy control programs [7]. The results of this policy are evidenced in the seven cases we reported in this study which received regular MDT after relapse.

We found that Colombian dapsone monotherapy patients can relapse. Further, in patients carrying *M*. *leprae* drug resistant mutations, current results show an association between drug resistance mutations and relapse (OR range 20.1–88.7, p < 0.05). This result suggests that dapsone monotherapy patients require focused attention because they have the potential to act as a reservoir for dapsone resistant strains [10, 11, 33]. However, it is difficult to identify at-risk individuals for relapse. For example, patients associated with leprosaria have been found to have higher frequency of dapsone resistance in this and prior studies, similar to that reported in Brazil [32,33]. However, no single department or town was a leprosy hot spot in this cross sectional surveillance.

Mutations outside of the genetic regions associated DR had been described in other reports [34, 35]. Uncharacterized polymorphisms and novel mutations have to be tested in vivo to test for resistance and add to our knowledge base. Phenotypic tests for drug resistance, such as those reported by Nakata et al [36], could be developed. Perhaps a saturated mutational analysis of target genes could be attempted to distinguish neutral polymorphisms from mutations leading to drug resistance [37,38].

Other targets for DR had been described, Monot et al [39] described *rpoT* for rifampicin, Singh et al [34] reported not clear explanation about rifampicin resistance phenotype of Airaku-3, a multi-drug resistance strain, the authors found two non-synonimous SNPs in transporter genes, *ctpC* and *ctpl*, however, a functional assay is required to determine if these variants confers any degree of rifampicin resistance.

This study had limitations and strengths. Due to sampling restrictions, conclusions regarding primary versus acquired resistance, and distinguishing relapse from reinfection, was not possible in the present study.

Current results are related with the initiative that WHO has formalized, an annual meeting for regions/countries serving as voluntary sentinel sites for leprosy drug resistance surveillance (DRS). The program has grown from its original focus on rifampicin resistance to molecular surveillance for dapsone and quinolone resistance [18]. The gene targets for quinolones are *gyrA* and *gyrB*, which encode *gyrA*ses A and B that are responsible for uncoiling DNA [37,40]. Reasons for including these targets are that ofloxacin is a standby drug for some patients and an alternative to the ROM (rifampicin, ofloxacin and minocycline) regimen. Moreover, fluoroquinolones are secondary drugs for TB [41]. An increase in availability and exposure to these drugs should be monitored for their potential to aid in the increase of drug resistant *M*. *leprae* [41,42].

The current study calls attention to *M*. *leprae* resistance in Colombia, especially the significant association between confirmed resistance mutations and relapse in leprosy patients. 70% of mutations we detected were related to drug resistance and were isolated from relapsed patients, five of which received dapsone monotherapy as a primary treatment. A high frequency of DRDR mutations for rifampicin was seen in a region where dapsone monotherapy was used extensively. The reported drug-resistance *M*. *leprae* suggests that the situation needs to be monitored in Colombia and the sentinel surveillance program have to continue in Colombia [32].
